# Liver at crossroads: unraveling the links between obesity, chronic liver diseases, and the mysterious obesity paradox

**DOI:** 10.1007/s10238-024-01493-y

**Published:** 2024-10-14

**Authors:** Maha Elsabaawy

**Affiliations:** Department of Hepatology and Gastroenterology, National Liver Institute, Menoufia University, Shebeen El-Kom, Egypt

**Keywords:** Obesity, Chronic liver disease, Non-alcoholic fatty liver disease, Obesity paradox, NAFLD, MAFLD, MASLD, Metabolic health, Cardiorespiratory fitness

## Abstract

Obesity is a global health issue that is intricately linked to the development and progression of chronic liver disease (CLD). This bidirectional connection, coupled with the obesity paradox (OP), presents a management dilemma. The established influence of obesity on the development and progression of chronic liver disease (CLD) is surpassed by the liver’s impact on the onset and advancement of obesity. Patients with CLD always experience increased energy expenditure, reduced appetite, and low protein synthesis, all of which might lead to weight loss. However, metabolic disturbances, hormonal imbalances, inflammatory signaling, immobility, drugs, and alterations in nutrient metabolism can contribute to the development and exacerbation of obesity. Despite the propagation of the OP concept, none of the guidelines has changed, recommending being overweight. Research bias and confounders might be the lifebuoy explanation. Additionally, overlooking the lethal morbidities of obesity for survival benefits full of suffering seems to be an illogical idea. Therefore, rather than endorsing an overweight status, emphasis should be placed on improving cardiorespiratory fitness and preventing sarcopenia to achieve better outcomes in patients with CLD. Accordingly, the complex interplay between obesity, CLD, and the concept of OP requires a sophisticated individualized management approach. Maximizing cardiorespiratory fitness and mitigating sarcopenia should be considered essential strategies for attaining the most favourable outcomes in patients with chronic liver disease (CLD).

## Introduction

**Fig. 1:**
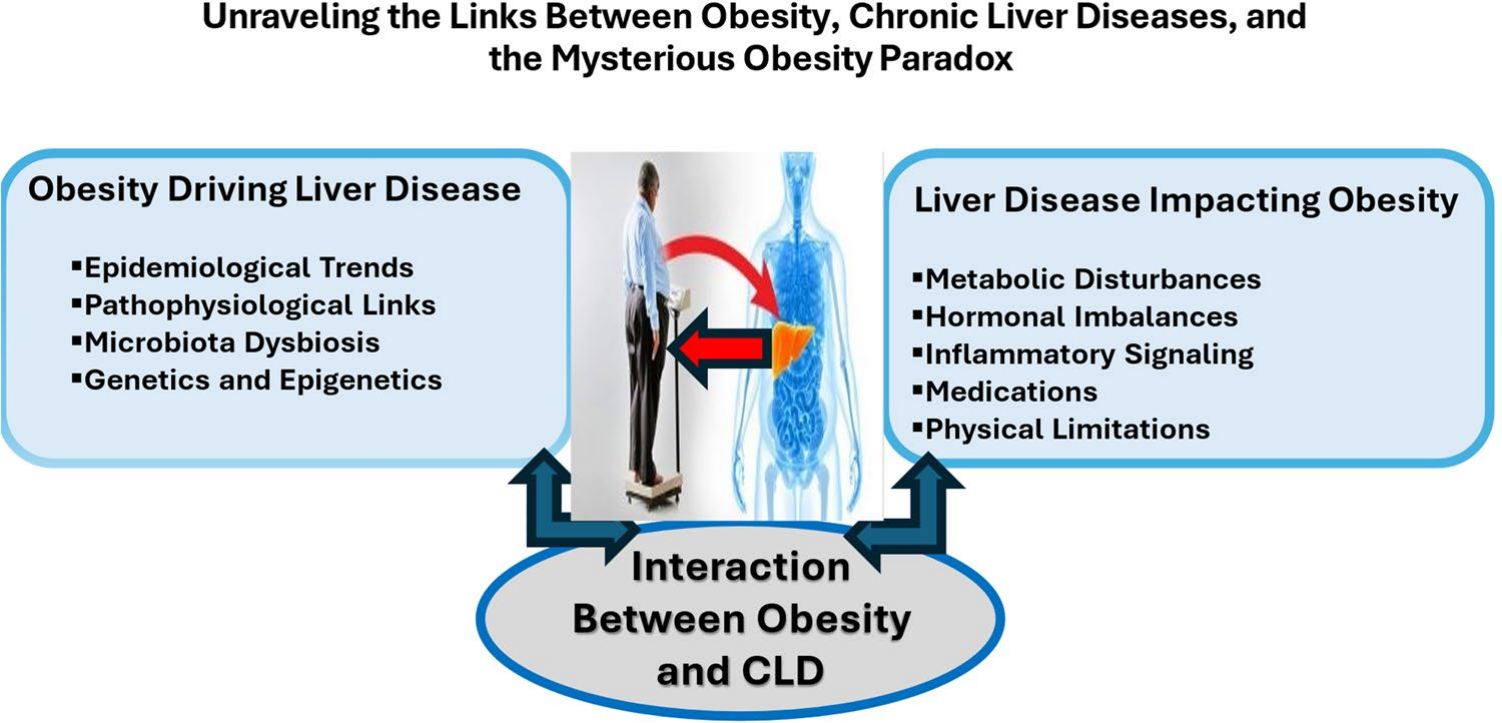
Interactions between obesity and chronic liver disease

The global increase in obesity has led to a surge in metabolic disorders, with chronic liver disease being a major outcome [1]. Non-alcoholic fatty liver disease (NAFLD), now termed metabolic-associated steatotic liver disease (MASLD), is the most prevalent liver disease worldwide [2]. Shared risk factors like genetics, diet, and a sedentary lifestyle exacerbate both obesity and liver disease, complicating the understanding of their cause-and-effect relationship [3]. The obesity paradox, where overweight or patients with obesity have better outcomes in some chronic liver diseases, is seen in liver cirrhosis, with studies indicating lower mortality rates for these patients compared to leaner individuals [4]. This challenges traditional views on the impact of body weight on disease progression [5] (Fig. 1).

### Impacts of obesity on liver disease

The complicated relationship between obesity and liver disease is a dynamic interplay that extends beyond a simple cause-and-effect paradigm.

## Obesity as a driver of liver disease

A lot of pathogenic mechanisms paved the way for the development of obesity-linked liver diseases:

### Epidemiological trends

The escalating prevalence of obesity has propelled a parallel surge in obesity-related liver diseases, most notably NAFLD [6]. Epidemiological studies have consistently demonstrated a strong association between obesity and NAFLD development, indicating that it is a prominent driver of liver pathology [7]. Obesity and NAFLD are more likely to progress to nonalcoholic steatohepatitis (NASH) and develop fibrosis, which can eventually lead to cirrhosis and liver failure [8].

### Pathophysiological links

The transition from obesity to liver disease involves complex metabolic and inflammatory events, with insulin resistance playing a central role by disrupting glucose and lipid metabolism [9]. Dysfunctional adipose tissue increases free fatty acids in the bloodstream, leading to hepatic lipid accumulation and steatosis [10, 11]. Dysregulated adipokines contribute to liver inflammation, with pro-inflammatory cytokines like TNF-α and IL-6 driving chronic inflammation [12, 13]. This inflammatory state accelerates the progression from steatosis to NASH, fibrosis, and cirrhosis [13].

### Hepatic lipotoxicity

Lipotoxicity, the harmful effects of lipid accumulation in non-adipose tissues, is particularly relevant in obesity-driven liver disease [14]. Excessive lipid deposition in the liver impairs hepatic function and induces oxidative stress [15]. Lipid peroxidation and the production of reactive oxygen species contribute to cellular damage, further fueling the inflammatory cascade and setting the stage for liver disease progression [16].

### Microbiota dysbiosis

Emerging research suggests that obesity-associated alterations in gut microbiota may play a role in the development of liver disease [17]. Dysbiosis, an imbalance in the composition and function of the gut microbiota, has been linked to increased gut permeability and translocation of bacterial products into the liver [18]. One mechanism by which dysbiosis may contribute to liver disease in obesity is the production of pro-inflammatory metabolites by certain gut bacteria. For example, increased levels of lipopolysaccharide (LPS) from gram-negative bacteria have been implicated in the development of NAFLD and NASH by promoting inflammation in the liver [19]. Furthermore, dysbiosis can affect the metabolism of dietary nutrients, leading to an imbalance in energy homeostasis and fat accumulation in the liver [20]. Short-chain fatty acids (SCFAs) produced by gut bacteria have been shown to influence hepatic lipid metabolism and may play a role in developing steatotic liver [21]. This phenomenon, known as the gut-liver axis, adds another layer of complexity to the relationship between obesity and liver disease, implicating the gut microbiome in the pathogenesis of NAFLD [22].

### Risk factors and modifiers (genetics and epigenetics)

Remarkably, individuals with varying genetic backgrounds are at a higher risk of developing chronic liver disease (CLD) in the context of obesity [23]. Accordingly, not all patients with obesity have MASLD, and many studies have identified certain genes and genetic polymorphisms as culprits in the occurrence and progression of MASLD in patients with obesity rather than others [24–27].

The PNPLA3 gene and Variants of the TM6SF2 gene encode a protein involved in lipid metabolism and are associated with an increased risk of developing NAFLD and its progression to more severe forms of liver disease, such as MASH and cirrhosis [28]. Studies have shown that certain variants of the PNPLA3 and TM6SF2 genes are linked to higher susceptibility to MASLD and adverse liver outcomes [29].

MBOAT7 encodes an enzyme involved in phospholipid metabolism and has been implicated in the development and progression of NAFLD [30]. Variants of MBOAT7 have been associated with an increased risk of NAFLD, NASH, and liver fibrosis. Research suggests that Genetic variations in MBOAT7 may influence lipid metabolism in the liver and contribute to the pathogenesis of NAFLD [30]. Additionally, a recent study identified the ABC1 (rs1800977) gene polymorphism CC genotype as a predictor of advanced fibrosis in patients with NAFLD [31].

#### Role of epigenetics

Epigenetics refers to changes in gene expression that do not involve alterations in the underlying DNA sequence. In patients with obesity who develop metabolic-associated fatty liver disease (MAFLD), epigenetic changes can play a significant role in disease progression [32, 33]. Changes in DNA methylation patterns can affect the expression of genes involved in lipid metabolism, inflammation, and insulin sensitivity, all of which are important factors in MAFLD development [34]. Additionally, alterations in histone modifications can influence gene expression related to metabolic processes and inflammation, thereby contributing to the development of MAFLD [35]. Furthermore, non-coding RNAs such as microRNAs can regulate gene expression post-transcriptionally and may play a role in the development of MAFLD by affecting key metabolic pathways [36].

To understand the claimed role of all these modifiers, we should imagine whether one individual is characterized as having a genetic predisposition to obesity. In contrast, another individual of a different ethnicity is illustrated as having a reduced risk of CLD, despite being overweight. A third individual, who maintains a healthy lifestyle through regular exercise and a balanced diet, is shown to have a lower risk of CLD than others in the group. This scenario symbolizes the complex interplay between genetics, epigenetics, ethnicity, and lifestyle factors in modulating the risk of CLD in the presence of obesity. Accordingly, while obesity is a major risk factor for CLD, this relationship is not uniform across all individuals. Genetic predisposition, ethnicity, and lifestyle factors can modify this risk.

## Obesity and progression of CLD

Obesity remains a central and complex factor in the progression of chronic liver disease. Its detrimental role in promoting liver inflammation, fibrosis, and cancer is well-established, particularly in the context of metabolic dysfunction [37]. However, the recent recognition of the obesity paradox, particularly in cirrhotic patients, underscores the need for a more nuanced understanding of how body composition impacts liver disease outcomes.

### Impact of obesity on cirrhosis and hepatocellular carcinoma

Once cirrhosis is established, obesity continues to play a role in disease progression but in more nuanced ways. Obesity is associated with an increased risk of decompensation events such as **ascites**, **variceal bleeding**, and **hepatic encephalopathy** due to heightened portal hypertension [38]. Additionally, individuals with obesity and cirrhosis are at an elevated risk of developing **HCC**. Studies have demonstrated that obesity, through chronic inflammation and insulin resistance, increases the likelihood of oncogenic transformation of hepatocytes [39]. In addition, obesity is associated with an increased risk of HCC development, even in the absence of liver cirrhosis [40].

### Obesity and treatment responses

Obesity is also a risk factor for poor outcomes of other forms of chronic liver disease, such as alcoholic liver disease, viral hepatitis, and drug-induced liver injury (DILI) [41]. In patients with chronic hepatitis C, obesity is associated with faster progression of liver fibrosis and a decreased response to antiviral therapy [42]. Obesity can also impact the response to treatments in CLD. In individuals undergoing liver transplantation for end-stage liver disease, obesity is associated with an increased risk of perioperative complications, including infections and delayed graft function [43]. However, post-transplant outcomes in patients with obesity have improved, partly due to better patient selection and management protocols [43].

In the context of pharmacological interventions, obese individuals with CLD often experience altered drug metabolism, necessitating careful dose adjustments to avoid under-treatment or toxicity [44]. Furthermore, weight loss interventions, including **bariatric surgery**, have shown promise in reversing or halting the progression of MAFLD/NASH, particularly when implemented early in the disease course [43]. However, these interventions must be balanced against the potential risks of rapid weight loss, which can exacerbate liver fibrosis in some cases.

## Obesity and mortality in CLD

The relationship between obesity and liver cirrhosis is complex. While obesity is generally considered a risk factor for various health issues, some studies have suggested that it may have a paradoxically positive impact on the survival of certain patients with liver cirrhosis. An exceptional concept named the obesity paradox (OP).

### Decoding the obesity paradox: a comprehensive review

The OP challenges conventional wisdom by suggesting that overweight or patients with obesity may experience better outcomes in certain chronic liver diseases compared to their lean counterparts [45]. This paradoxical relationship raises compelling questions about the intricate interplay between adiposity and liver health, challenging traditional viewpoints and prompting a nuanced exploration of the underlying mechanisms.

**Fig. 2:**
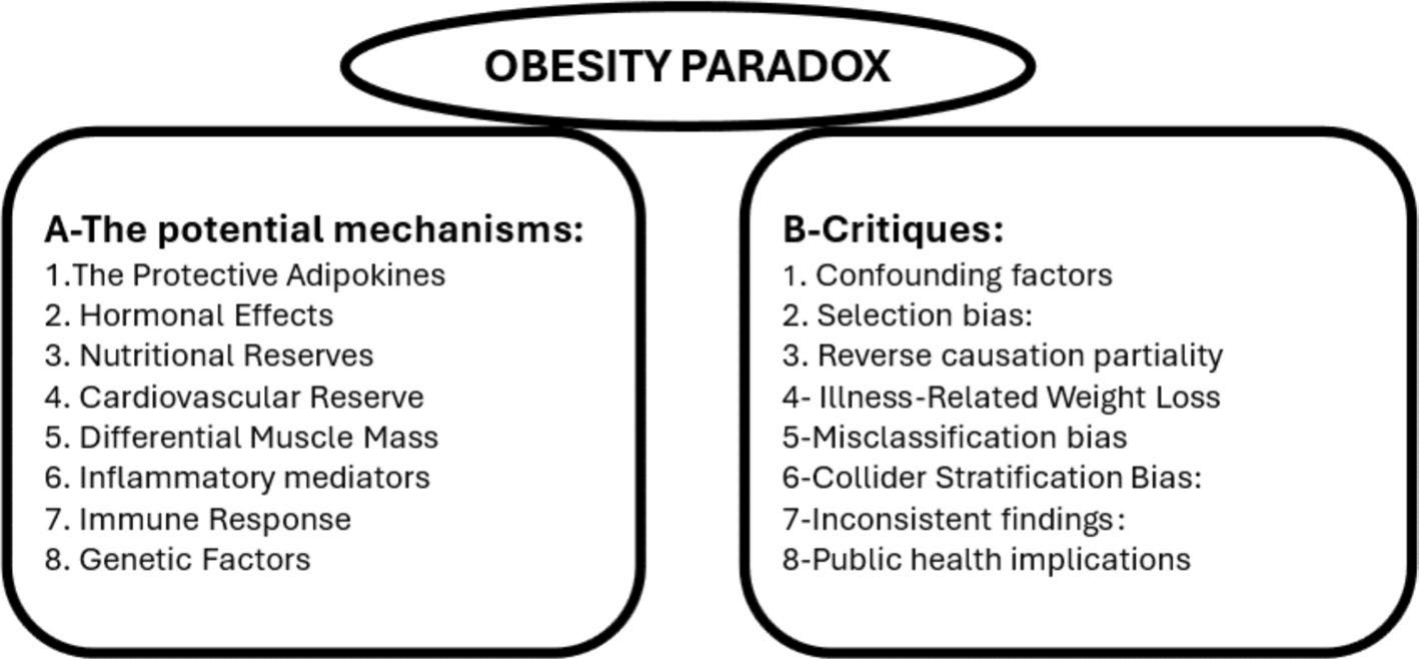
Obesity paradox: theories and critiques

OP in the context of liver diseases presents a complex narrative wherein overweight or patients with obesity, despite their heightened risk for developing hepatic conditions, exhibit surprising survival advantages compared to their lean counterparts [46]. In a study by Kargozian et al., clinical outcomes, including mortality, length of stay, and total hospital charges, were compared between hospitalized with or without obesity cirrhotic patients. The findings supported the OP observed in other intensive care patients, revealing a decrease in inpatient mortality associated with obesity but with higher costs and prolonged hospitalization in cirrhotic patients [47]. The Kargozian study was limited by its retrospective design, biases, and missing anthropometric and BMI data [47]. A recent retrospective observational study evaluated the impact of obesity on survival in 1180 chronic liver disease patients over 2.5 years. The analysis showed that obesity, especially in patients aged 65 and older, was associated with better overall survival [48]. Despite the limitations, it was found that lower BMI predicted higher mortality and shorter survival in hepatocellular carcinoma patients. Furthermore, 20% of these patients were undernourished, which might have affected the results since malnutrition is a poor prognostic factor in terminally ill patients [49]. Available data suggest that obesity before liver disease onset can be harmful, but a higher BMI in advanced liver disease may indicate a better prognosis [50]. Thus, fat mass may be associated with better nutritional status and improved survival, though it doesn’t guarantee better outcomes [49] (Fig. 2).

## The potential mechanisms underlying the obesity paradox

Unravelling the enigma of OP requires an exploration of potential mechanisms. Here are some suggested theories:

### The protective adipokines and hormonal effects

Beyond storing fat, adipose tissue is an active endocrine organ that secretes various adipokines and hormones. Some of these, such as leptin and adiponectin, might confer protective cardiovascular and metabolic effects. Leptin plays a role in energy regulation and might help mitigate some of the adverse effects of chronic diseases by modulating inflammation and immune response [51]. Higher levels of adiponectin, despite being lower in patients with obesity, can improve insulin sensitivity and exert anti-inflammatory properties, potentially offsetting some negative health outcomes associated with obesity [52]. Obesity and hypertension are significant risk factors for cardiovascular diseases, impacting millions globally. Both conditions are linked to chronic low-grade inflammation, driven by adipokines like adiponectin [53]. As the most prevalent adipokine, adiponectin benefits metabolic and vascular health, although elevated serum levels are associated with certain syndromes. The “adiponectin paradox” remains unclear in the context of obesity-related hypertension. Ultimately, in cases of obesity-related hypertension, compensatory mechanisms, adiponectin resistance, and reduced adiponectin clearance—stemming from impaired kidney and liver function—occur together, leading to what is known as the “adiponectin paradox” [53].

### Nutritional reserves

Obesity provides greater nutritional reserves in the form of fat and lean body mass, which can be advantageous during acute or chronic illnesses [54]. These reserves can be particularly beneficial during periods of severe infection, when the body experiences increased metabolic demands and catabolism. In such scenarios, having additional energy stores might improve survival by preventing malnutrition and muscle wasting, critical risk factors for poor outcomes in hospitalized patients [54].

### Cardiovascular reserve

Patients with obesity often develop a greater cardiovascular reserve due to the increased workload imposed on the heart by excess weight [55]. This adaptation might provide a protective buffer during periods of stress or illness. The larger heart size and increased cardiac output could improve the ability to maintain adequate perfusion and oxygen delivery during critical illnesses [56].

### Differential muscle mass

Obesity is often accompanied by increased muscle mass, which can be protective in chronic diseases. Greater muscle mass is associated with better physical function and metabolic health, which may contribute to improved survival [57]. Muscle acts as a reservoir of amino acids necessary for immune function and wound healing. In times of illness, the presence of greater muscle mass can help maintain essential bodily functions and support recovery processes [58].

### Inflammatory and immune response

While chronic inflammation is a hallmark of obesity, the inflammatory response in patients with obesity might play a dual role [59]. Low levels of chronic inflammation might prime the immune system, potentially resulting in a more effective response to acute infections or stressors [60].

### Genetic factors

Genetic factors might influence both obesity and survival outcomes in chronic diseases [61]. Certain genetic variants associated with obesity might also confer a survival advantage in specific contexts [61]. These genetic factors could affect how individuals respond to illness, their propensity for developing complications, and their overall resilience to health challenges [62].

## Obesity paradox critiques

Numerous meta-analyses have investigated OP, prompting certain researchers to assert that the remarkable coherence of the data leaves little room for doubt, suggesting that these observational results are more than mere statistical artifacts and instead reflect biological credibility [63–71]. However, this is still a matter of controversy and raises questions about the logic and certainties of this unacceptable concept. An increasing volume of research has delved into the potential methodological factors contributing to OP. Some examples of methodological bias are as follows:

### Confounding factors

Many studies that report OP fail to account for confounding variables, such as age, sex, smoking status, and underlying health conditions. These factors can influence the relationship between weight and health outcomes, leading to biased results [72]. Age and Comorbidities: Older adults with obesity might have different health outcomes compared to younger individuals. Comorbid conditions like diabetes or hypertension can also influence mortality rates and health outcomes, making it difficult to isolate the effect of obesity itself. Factors such as income, education, and access to health care can confound results. For instance, individuals with higher socioeconomic status may have better overall health despite being classified as patients with obesity.

### Selection bias

Selection bias occurs when individuals included in a study are not representative of the broader population, leading to skewed or inaccurate results [73]. In the context of OP, selection bias can occur if only certain subgroups of the population are included in the analysis, such as excluding individuals with severe obesity or significant comorbidities [74]. For example, if a study only included patients with a specific type of heart disease and excluded those with other cardiovascular conditions, the findings may not apply to a broader population of individuals with heart disease. This can lead to an overestimation of the protective effects of obesity, as the study results may not accurately reflect the true relationship between weight and health outcomes in the general population. Selection bias can also occur if individuals are selectively included or excluded based on certain characteristics such as age, gender, or socioeconomic status. If these factors are related to both weight and health outcomes, the results of this study may be biased [73].

### Reverse causation partiality

Reverse causation is a phenomenon in which the relationship between two variables is not as straightforward as it may appear, and the direction of causality is reversed from what is initially assumed [75]. In the context of OP, reverse causation suggests that the observed association between higher BMI and better health outcomes may be due to factors other than weight, such as underlying health conditions or illness leading to weight loss [75]. Critics argue that the OP may be a result of reverse causation, where individuals who are underweight or have a lower BMI may be sicker or have underlying health issues that contribute to their poorer outcomes rather than their weight itself. For example, researchers have been studying the link between obesity and diabetes. They might incorrectly assume that obesity causes diabetes, but diabetes leads to weight gain, which can lead to obesity. That is reverse causation—mixing up the cause and effect. Consider a study that examines the relationship between BMI and mortality in older adults. Individuals with a lower BMI are more likely to have pre-existing health conditions such as cancer, heart disease, or chronic obstructive pulmonary disease (COPD). These conditions could drive the association between lower BMI and increased mortality, rather than weight being the cause.

### The overlooked illness-related weight loss

This bias refers to weight loss due to illness, which can distort the association between weight status and outcomes. In studies where weight is measured at one point in time (baseline) and participants are followed for outcomes, individuals who experience weight loss due to illness may erroneously appear to have a lower risk of adverse outcomes because they are classified as having a lower weight. This can create the illusion that being overweight or with obesity is protective when, in fact, weight loss due to illness influences the results [76]. In another example, individuals who have experienced significant unintentional weight loss due to illness or malnutrition may appear to have lower BMI and worse health outcomes. In this case, weight loss is a consequence of underlying health issues, rather than weight itself being the primary factor influencing the outcomes.

### Misclassification bias stemming from BMI as an obesity metric

Relying solely on BMI as the standard measurement tool for obesity overlooks the variations in body composition. Instead, more accurate measurements, such as cardiorespiratory fitness, and methods such as the abdomen-to-hip ratio should be considered [77]. Enhanced cardiorespiratory fitness (CRF) improves CVD outcomes across different BMI groups, altering the obesity paradox in patients with heart failure (HF) and coronary heart disease (CHD) patients [77–79].

### Collider stratification bias

This bias arises when conditioning on the common effect of two variables (a “collider”) introduces an association between them. In the context of the obesity paradox, consider a scenario in which both obesity and smoking are risk factors for diseases such as renal cell carcinoma (RCC), which is associated with mortality. If researchers only adjust for RCC when examining the association between obesity and mortality, it might inadvertently induce collider stratification bias. This bias occurs because patients with obesity may be less likely to smoke (as smoking and obesity are inversely related) and therefore appear to have better survival rates, not because obesity is protective but because it is indicative of not smoking, which is a stronger risk factor for mortality. [80]. A collider stratification bias occurs when a variable, known as a collider, is conditioned upon in statistical analyses, leading to biased results. In the context of the obesity paradox, collider stratification bias can occur when researchers stratify their analyses based on a variable that is affected by both exposure (obesity) and outcome (health outcomes), leading to distorted associations [80]. For example, let us consider a study that examined the relationship between obesity and mortality in individuals with heart disease. Researchers may stratify their analyses based on variables such as hospitalization. In this case, hospitalization is a collider because it is influenced by both obesity and severity of heart disease. Accordingly, this study may produce distorted results that falsely suggest a protective effect of obesity on mortality or mask its true impact. To mitigate collider stratification bias in the context of OP, researchers should carefully consider which variables they condition their analyses on and ensure that they do not inadvertently create bias by conditioning on colliders. Additionally, employing appropriate statistical methods such as structural equation modeling or directed acyclic graphs can help identify and address potential collider bias in observational studies.

### Inconsistent findings

While some studies have suggested that individuals with a higher BMI may have better outcomes in certain health conditions, other studies have contradicted these findings or have not found evidence to support the obesity paradox. One example of conflicting evidence comes from a study by Bagheri et al., who conducted a meta-analysis of survival after renal cell carcinoma (RCC) and found that patients with obesity and RCC had higher cancer-specific survival but lower overall survival. The authors argue that the finding of lower overall survival in patients with obesity contradicts the notion of an obesity paradox, rather than creating a new “paradox within a paradox.” [81]. Conversely, Mazimba et al. identified an “overweight paradox” in pulmonary hypertension, where overweight patients had the lowest 5-year survival rates, while normal and underweight patients had the highest [82]. Another study by Inagaki et al. found that a higher BMI was associated with an increased mortality risk among Japanese hemodialysis patients. This study suggested that the obesity paradox observed in some populations may not apply to individuals with end-stage renal disease [83]. These examples reveal that the relationship between obesity and survival is complex and disease-specific, challenging the generalized concept of OP. This inconsistency in findings raises questions about the generalizability of OP and its applicability to diverse patient groups.

### Public health implications

Promoting the idea of the OP may send conflicting messages about the risks of obesity and potentially undermine efforts to address the obesity epidemic through prevention and intervention programs.

The OP concept highlights the need for careful methodological design in research. Addressing potential biases such as selection bias, confounding factors, and reverse causation is crucial for accurately interpreting the relationship between obesity and health outcomes. Future studies should aim to use longitudinal designs, adjust for confounding variables, and consider individual patient characteristics to provide a clearer understanding of this complex issue.

## Impacts of liver disease on obesity

Chronic liver disease can also have a significant impact on the occurrence and progression of obesity. Patients with CLD always experience increased energy expenditure, reduced appetite, and low protein synthesis, all of which may lead to weight loss [84–86]. However, metabolic disturbances, hormonal imbalances, inflammatory signaling, immobility, drug side effects, and alterations in nutrient metabolism can contribute to the development and exacerbation of obesity in patients with CLD.

### Metabolic disturbances

Chronic liver diseases, such as NAFLD and NASH, impair the liver’s ability to metabolize nutrients like fats and carbohydrates, leading to disruptions in energy metabolism, insulin resistance, and dyslipidemia, which can contribute to weight gain and obesity [87]. In liver cirrhosis, altered nutrient metabolism can disrupt energy balance, with impaired glycogen storage and gluconeogenesis causing blood glucose fluctuations, increased appetite, and overeating [88, 89]. The compromised liver function also hampers lipid metabolism, leading to triglyceride accumulation, particularly in visceral fat, predisposing individuals to obesity [90]. Additionally, liver cirrhosis increases energy expenditure due to the metabolic demands of the diseased liver and complications like ascites and hepatic encephalopathy, which, combined with nutrient metabolism impairment, can cause an energy imbalance and weight gain [87–93].

### Hormonal imbalances

Liver diseases can disrupt the production and regulation of hormones, which play a role in metabolism and appetite control [92]. For example, liver dysfunction can lead to alterations in the levels of leptin, ghrelin, and adiponectin, hormones involved in regulating hunger, satiety, and energy balance [93]. These hormonal imbalances contribute to overeating, weight gain, and obesity.

### Inflammatory signaling

Inflammation associated with liver disease can contribute to systemic inflammation, which is implicated in the development of obesity [94]. The release of pro-inflammatory cytokines from the liver may influence adipose tissue function and contribute to weight gain [95].

### Medications and treatments

Patients with chronic liver disease may be prescribed medications or undergo treatments that can cause side effects related to weight gain. Some medications used to treat liver disease or its complications that may cause weight gain as a side effect include:

Corticosteroids are commonly prescribed for conditions such as autoimmune hepatitis and to reduce inflammation in liver diseases such as hepatitis. These medications can cause weight gain because they affect metabolism, appetite stimulation, and fluid retention [96].

Some antipsychotic medications, such as olanzapine and clozapine, are used to manage psychiatric symptoms in patients with liver diseases. These medications can lead to weight gain through mechanisms such as increased appetite, metabolic changes, and altered fat distribution [97].

Certain antidepressants, including tricyclic antidepressants and selective serotonin reuptake inhibitors (SSRIs), may be used to treat mood disorders in patients with liver disease, but they can cause weight gain due to changes in appetite, metabolism, and hormonal balance [98]. Additionally, medications like thiazolidinediones (TZDs), prescribed to improve insulin sensitivity in conditions like NASH or diabetes, can also lead to weight gain, often due to fluid retention and increased fat storage [99]. Moreover, anticonvulsants such as valproic acid or carbamazepine, used for seizure management, can contribute to weight gain through mechanisms like increased appetite, metabolic changes, and altered energy balance [100].

### Physical limitations

In advanced stages of chronic liver disease, such as cirrhosis, physical limitations and reduced mobility can lead to a sedentary lifestyle and decreased physical activity, contributing to obesity [101]. Sarcopenia, often seen in chronic liver disease, exacerbates obesity in critically ill patients [102]. Sarcopenic obesity (SO) presents a dual challenge in cirrhotic patients, combining muscle wasting and excess adiposity, which worsens physical function, quality of life, and metabolic complications [103]. Managing SO requires a comprehensive approach, including nutritional support and physical rehabilitation, to address both muscle loss and fat accumulation [104]. Assessing sarcopenia in obese liver disease patients can be challenging due to excess fat and water retention, with CT and MRI being the most accurate methods [105]. Sarcopenic obesity in cirrhotic patients is associated with increased mortality, sepsis, hyperammonemia, hepatic encephalopathy, and extended hospital stays post-transplantation [86, 105–107].

Importantly, myosteatosis, or fat infiltration in skeletal muscles, often accompanies sarcopenia, exacerbating muscle loss and metabolic issues, further complicating disease progression [54].

## Defining obesity in patients with CLD

In patients with CLD, traditional anthropometric measures such as BMI are unreliable due to the frequent coexistence of sarcopenia (loss of muscle mass) and dynapenia (reduced muscle strength) [108]. These conditions, common in advanced liver disease, distort BMI’s ability to accurately reflect metabolic health [108]. Sarcopenic obesity and dynapenic obesity complicate the clinical picture [53]. Moreover, BMI is often skewed by fluid retention in CLD, making it an inadequate measure of obesity or body composition [108].

### The cardiorespiratory fitness conception as a lifebuoy

Given these limitations, cardiorespiratory fitness (CRF) has emerged as a superior marker for assessing metabolic and functional health. CRF, often measured through maximal oxygen uptake (VO2 max), evaluates the ability of the circulatory and respiratory systems to supply oxygen to the muscles during sustained physical activity [109]. CRF stands as a robust and consistent predictor of both morbidity and mortality in adults, as demonstrated by an extensive review of meta-analyses encompassing over 20.9 million observations from 199 distinct cohort studies [110]. Elevated CRF is linked to a reduced risk of cardiovascular disease (CVD). Moreover, evidence indicates that CRF can mitigate the relationship between BMI and CVD outcomes [111]. There is substantial evidence linking BMI, waist circumference, and CRF, with elevated obesity levels generally correlating with diminished CRF [112, 113]. The interplay between obesity and CRF collectively influences both cardiovascular disease (CVD) risk and prognosis [114]. Notably, higher CRF can attenuate the CVD risk associated with obesity, as studies have demonstrated improved outcomes in obese individuals possessing elevated CRF [114]. Unlike BMI, CRF reflects the patient’s ability to withstand the catabolic stress of liver disease and guides therapeutic strategies like exercise interventions to improve fitness and clinical outcomes [114]. Some studies suggest that individuals who are overweight or with obesity but have higher levels of CRS may have better health outcomes with a decrease in all-cause mortality than those who are normal weight but have lower fitness levels [115–118]. This could be due to the protective effects of physical activity and exercise on various health parameters regardless of body weight [115].

CRF can be measured either directly as maximal oxygen consumption (Vo2max) or estimated from peak work rates on treadmills, cycle ergometers, or nonexercised algorithms [111]. Although Vo2max is more accurate, the estimated CRF is more commonly used in large epidemiological studies because of its practicality. Both measured and estimated CRF are independent predictors of health outcomes [118].

Improving cardiorespiratory fitness (CRF) is crucial for patients with CLD, as it has several benefits. Enhanced Metabolic Health: Regular aerobic exercise can improve insulin sensitivity, reduce liver fat, and ameliorate inflammation. While weight loss may be beneficial, improving CRF can lead to better health outcomes regardless of changes in body weight. Increased CRF enhances overall physical function, which is particularly important for patients with chronic diseases who may be at risk for sarcopenia.

Accordingly, CRF might be the Savior from the dilemma of OP with the recommended implementation of surveillance programs for CLD and cirrhotic patients for better prognostication and risk stratification.

### Clinical implications and treatment considerations

Based on the critiques of OP and conflicting evidence from various studies, it is important to approach recommendations regarding BMI and health outcomes with caution. The following are some considerations and recommendations based on critiques.

1. *Fitness levels* Cardiorespiratory fitness plays a critical role in overall health. Individuals with higher fitness levels, regardless of weight, often experience better outcomes. Therefore, focusing on improving fitness rather than solely on weight loss may be more beneficial.
2. *Individualized assessments* Each patient presents a unique profile influenced by genetics, lifestyle, comorbidities, and personal goals. Tailoring interventions based on these factors allows for more effective management strategies.
3. *Sarcopenia considerations* Recognizing the risk of sarcopenia in both obese and non-obese individuals is crucial. Interventions should aim to preserve or enhance muscle mass through resistance training and adequate nutrition, which can mitigate the risks associated with both obesity and chronic diseases.
4. *Holistic health perspective* A comprehensive approach that incorporates physical activity, nutrition, mental health support, and regular monitoring of metabolic health can lead to improved outcomes for patients across the weight spectrum.
5. *Consider context* Knowledge that OP may not apply universally to all populations or disease conditions. When interpreting research findings or making recommendations, the specific context, study design, and population characteristics should be considered to understand the implications for different groups of individuals.
6. *Monitor health metrics* Regular monitoring of relevant health metrics, such as blood pressure, cholesterol levels, blood glucose, and waist circumference, with special consideration of CRF, can provide a more comprehensive picture of an individual’s health status beyond BMI. These metrics can help guide healthcare recommendations and interventions tailored to specific health needs.
7. *Consult healthcare professionals* Seeking guidance from healthcare professionals, such as physicians, dietitians, and other experts in the field of obesity research to develop personalized recommendations based on current evidence and best practices.

In managing the intricate relationship between obesity, CLD, and OP, focusing solely on weight status is insufficient. A paradigm shift towards improving cardiorespiratory fitness and preventing sarcopenia offers a more effective strategy for enhancing overall health outcomes in these patients. By adopting a holistic approach that prioritizes physical fitness and muscle preservation, healthcare providers can significantly improve the quality of life and prognosis for individuals affected by these interconnected conditions.

**Future research** must be directed to the impact of weight loss on patients with obesity and CLD, better diagnostics of adiposity in compensated and decompensated liver cirrhosis patients, well-designed robust studies investigating OP alleges, and studies analyzing the short- and long-term impacts of fair CRF on the well-being of patients with CLD, even in the presence of obesity. As research continues to unravel the complexities of the bidirectional relationship between obesity and liver disease, future perspectives will include a focus on precision medicine, personalized interventions, and innovative therapeutic modalities.

**Conclusively**, the management of obesity-related chronic liver disease involves lifestyle changes, medications, and, in severe cases, bariatric surgery. However, the presence of the obesity paradox raises questions regarding the best approach to weight management in patients with established liver disease. Although the obesity paradox suggests better survival for patients with obesity and chronic disorders, it is important to consider the high number of related health issues experienced by these patients and their communities. Therefore, focusing on the absence of sarcopenia and fair cardiorespiratory fitness may guarantee better outcomes in chronic diseases, even in the presence of obesity.

## Data Availability

No datasets were generated or analysed during the current study.

## Abbreviations

NAFLD: Non-alcoholic fatty liver disease
MAFLD: Metabolic-associated fatty liver disease
NASH: Non-alcoholic steatohepatitis
MASLD: Metabolic-associated steatotic liver disease
CLD: Chronic liver disease
BMI: Body mass index
SO: Sarcopenic obesity
OP: Obesity paradox
DILI: Drug-induced liver injury
CRF: Cardiorespiratory fitness
CVD: Cardiovascular disease
